# SAA1 knockdown promotes the apoptosis of glioblastoma cells via downregulation of AKT signaling

**DOI:** 10.7150/jca.48419

**Published:** 2021-03-10

**Authors:** Huikai Zhang, Yang Xu, Gang Deng, Fanen Yuan, Yinqiu Tan, Lun Gao, Qian Sun, Yangzhi Qi, Kun Yang, Rongxin Geng, Hongxiang Jiang, Baohui Liu, Qianxue Chen

**Affiliations:** 1 Department of Neurosurgery, Renmin Hospital of Wuhan University, Wuhan, China.; 2 Central Laboratory, Renmin Hospital of Wuhan University, Wuhan, China.

**Keywords:** Serum amyloid A1, Glioblastoma, Apoptosis, AKT, Temozolomide.

## Abstract

Serum amyloid A1 (SAA1) is an inflammatory associated high-density lipoprotein. And It is also considered as a predictor and prognostic marker of cancer risk. However, its role and mechanisms in glioblastoma (GBM) still unclear. In this study, we validate that SAA1 is up-regulated in GBM, and its high expression predicts poor prognosis. SAA1 knockdown promotes the apoptosis of GBM cell. Mechanistically, SAA1 knockdown can inhibit serine/threonine protein kinase B (AKT) phosphorylation, thereby regulating the expression of apoptosis-related proteins such as Bcl2 and Bax, leading to GBM cell death. Moreover, Gliomas with low SAA1 expression have increased sensitivity to Temozolomide (TMZ). Low SAA1 expression segregated glioma patients who were treated with Temozolomide (TMZ) or with high MGMT promoter methylation into survival groups in TCGA and CGGA dataset. Our study strongly suggested that SAA1 was a regulator of cells apoptosis and acted not only as a prognostic marker but also a novel biomarker of sensitivity of glioma to TMZ.

## Introduction

Glioma is the malignant tumor with the highest incidence in the central nervous system. Its incidence is second only to meningiomas, accounting for about 40% to 60% of intracranial tumors 1. Among gliomas, glioblastomas account for the highest proportion. Gliomas usually show a high degree of cleavage, necrosis, and aggressiveness 2. At present, most glioma treatment methods are surgical treatment, and then decide whether to perform radiochemotherapy according to the tumor pathological grade and molecular diagnosis of the patient 3. However, due to the high invasiveness of the tumor, it is difficult to completely remove. Despite aggressive surgical and routine treatment, the average survival time of patients after diagnosis is only 12-15 months 4. At present, more and more molecular biology studies on gliomas have given scholars a deeper understanding of the pathogenesis of such tumors. Gene therapy has gradually become a research hotspot, and search for biomarkers that can detect early, diagnose early, and monitor prognosis and recurrence is a priority.

SAA1 is a sensitive acute phase high-density lipoprotein, which is mainly produced by the liver as a response to the body's acute inflammatory and tissue damage. It is generally used to evaluate the course of acute phase reactions. SAA1 may participate in the body's immune system, promote the repair of damaged tissues, and be used as a diagnostic or prognostic marker for many diseases 5, 6. In recent years, the role of SAA1 in the occurrence and development of tumors has received increasing attention. It is considered as a predictor and prognostic marker of tumor risk 7, 8. It plays a significant role in the malignant progression and prognosis of many tumors. In patients with prostate cancer, gastric cancer, lung cancer, breast cancer, endometrial cancer, esophageal cancer and melanoma, the high expression of serum SAA1 levels have been shown to be associated with poor prognosis and cancer aggressiveness 9-15. In uterine cancer, SAA1 levels increase as the disease progresses and may be a candidate serum biomarker 16; Non-small cell lung cancer patients who treated with EGF receptor tyrosine kinase inhibitors with elevated serum SAA1 levels have a poor prognosis 17; At the same time, because chronic inflammation is closely related to malignant transformation, SAA1 may affect tumorigenesis and metastasis.

Increasing evidence indicates that increased expression of SAA1 may also be related to the mechanism of tumorigenesis and development 18. Some special SAA1 subtypes such as SAA1.1 and SAA1.3 can inhibit tumor metastasis by inducing tumor cell apoptosis and inhibiting angiogenesis 19; SAA1 is mediated by activating ERK and formyl peptide receptor-like-1 (FPRL1) Signaling pathways to increase expression of matrix metalloproteinases in monocytes, regulate inflammation and immune responses and promote metastatic growth of tumor cells^20^; SAA1 can also promote tumor cell migration by inducing adhesion molecule expression, angiogenesis, and matrix degradation 21. Knebel et al found that astrocytoma patients (AGII to AGIV/GBM) have increased levels of serum SAA and *SAA1* mRNA and protein levels are increased in GBM brain tissue 22. In addition, recombinant SAA1 can induce cell proliferation, produce nitric oxide (NO) and reactive oxygen species (ROS), make a positive effect to the migration and invasion of T98G and A172 cell lines, expression activity of matrix metalloproteinases 2 and 9 (MMPs), and IL-8 secretion^23^. Although the role and mechanisms of SAA1 in GBM are still unclear, previous research speculates SAA1 may influence tumor progression by participating in a molecular network that connects inflammation, cell proliferation and angiogenesis.

AKT is a protein kinase that regulates cell proliferation, metabolism, and protein synthesis under the action of different cell growth factors and external stimuli 24, 25. Furthermore, some studies have found that the AKT cascade mechanism affects tumor invasion and progression in a variety of human cancers by varying degrees of activation 26, 27. AKT is involved in various signaling pathways that regulate proteins associated with cancer phenotypes, such as apoptosis-related proteins Bcl2 and bcl-2 cell death antagonist (BAD), to prevent apoptosis. Ligands such as growth factors, cytokines, hormones and mitogens bind to cell membrane receptors to trigger AKT activation 28. Some studies have found that AKT is also critical for brain development and the viability of malignant glioma cells 29.

In this study, we discovered a new role of SAA1 in regulating GBM cell apoptosis. We demonstrated that depletion of SAA1 could induce GBM cell apoptosis. Mechanistically, SAA1 knockdown can inhibit AKT phosphorylation, thereby inhibiting Bcl2 and promoting Bax expression, leading to GBM cell death. Meanwhile, the expression level of SAA1 may be related to the sensibility to TMZ in GBM patients.

## Materials and methods

### Bioinformatics

To clarify the expression and prognostic role of SAA1 in gliomas, we used the Gene Expression Omnibus (GEO) dataset (https://www.ncbi.nlm.nih.gov/gds/), Gliovis database(http://gliovis.bioinfo.cnio.es/) and the UCSC Xena platform (http://xena.ucsc.edu/). GSE52009 was downloaded from the GEO; the data about mRNA expression, survival analysis and Pearson correlation analysis were from The Cancer Genome Atlas (TCGA) and Chinese Glioma Genome Atlas (CGGA) database which downloaded in the Gliovis. Specific information on postoperative treatments (chemo/radiotherapy) of glioma patients was downloaded from UCSC Xena platform. Making analysis about biological mechanism related to SAA1 expression by Gene set enrichment analysis (GSEA).

### Clinical tissue samples

Glioma tissues were obtained from the department of neurosurgery in Renmin hospital of Wuhan University, Wuhan, China. None patients received any chemo-or radiotherapy before surgery. Non-glioma tissues were collected from patients with severe traumatic brain injury during surgery and informed consent was obtained from the patients and their families. All patients signed informed consents and this study received the approval of the Ethics Committee of Renmin Hospital of Wuhan University (approved number: 2012LKSZ (010) H.

### Immunohistochemical staining

The paraformaldehyde-fixed paraffin tissue microarray that contained 140 glioma tissues was used. The microarray was incubated with a primary anti-SAA1 monoclonal antibody (YN3036, immunoway, USA) overnight at 4°C. Images were captured using an Olympus BX40 microscope (Tokyo, Japan). The result was primarily based on the strength of staining and the number of positive cells. 10 high magnification fields were randomly selected for observation.

### ELISA assay

The ELISA kit (HM10751, Bio-Swamp, China) assay Human SAA1 level in the sample, used purified Human SAA1 antibody to coat microtiter plate wells, made solid-phase antibody, then added SAA1 to wells. Combined SAA1 antibody with HRP labeled, became antibody-antigen-enzyme-antibody complex, added TMB substrate solution, TMB substrate became blue color at HRP enzyme-catalyzed, reaction was terminated by the addition of a sulphuric acid solution and the color change was measured at a wavelength of 450 nm. The concentration of SAA1 in the samples was then determined by comparing the O.D. of the samples to the standard curve.

### Antibodies and drugs

The antibodies included the following: anti-SAA1 (YN3036, immunoway, USA), anti-GAPDH (#5174, Cell signaling Technology(CST), USA), anti-Bcl-2 (127891, Proteintech, USA), anti-Bax (50599-2, Proteintech), anti-Phospho-AKT(#4060, CST), anti-AKT (#4691, CST), anti-cleaved-caspase3 (ab32042, Abcam, UK), anti-caspase3 (19677-1-AP, Proteintech). AKT activator SC79 was purchased form Sellect (S786303, purity (>97%), USA) and dissolved in dimethyl sulfoxide (DMSO), which was obtained from Servicebio (G5051, Wuhan, China).

### siRNA transfection

SiRNA that specific targeting human SAA1 mRNA (SiSAA1-1, SiSAA1-2) and negative control SiRNA (SiNC) were obtained from RiboBio Corporation (Guangzhou, China). Transfection was done using X-tremeGENE SiRNA transfection reagent (Roche, Germany) according to the manufacturer's protocol. The sequences included the following:

SiSAA1-1: GCGATGCCAGAGAGAATAT (Target sequence)

SiSAA1-2: CTGGCCTGCCTGAGAAATA (Target sequence)

### Cells, cell culture and transfection

U251 and U87 which are two human glioblastoma-derived cancer lines, were purchased from the Cell Bank Type Culture Collection of the Chinese Academy of Sciences (Shanghai, China). Cell lines were all cultured at 37°C under a humidified atmosphere of 5% CO2 by using Dulbecco's modified Eagle's medium (DMEM) supplemented with 10% fetal bovine serum (FBS) (Gibco, Invitrogen, Carlsbad, CA, USA). SC79 was used at 10 µM.

### Flow cytometric analysis

Annexin V-PE/7-AAD kit (Becton Dickinson, USA) were used to measure the apoptosis of glioma cells. As request of the manufacturer's instruction, Cells were digested and collected, washed for 3 times, stained with Annexin VPE/7-AAD for 10 min in the dark. Apoptosis was analyzed by FACS Calibur flow cytometer (Becton Dickinson). PE Annexin V and 7-AAD negative indicate viable; PE Annexin V positive and 7-AAD negative indicate early apoptosis; PE Annexin V and 7-AAD positive indicate late apoptosis or dead. Early apoptosis and late apoptosis were summed and the total apoptosis rate was calculated.

### Mitochondrial membrane potential (∆Ψm) assay

JC-1 fluorescent probe (Yeasen, Shanghai, China) was used to detect the change of ∆ψm. Images were obtained by Olympus BX51 microscope (Olympus, Japan). The change of ∆ψm was reflected by the change of fluorescence color caused by the change of JC-1 morphology from aggregates to monomers.

### Western blotting

U251 and U87 were lysed in a modified RIPA buffer (Beyotime, Shanghai, China) on ice for about 30 minutes, then centrifuged at 12,000rpm for 15 minutes. For frozen glioma tissues, we added 1ml of RIPA lysate per 100mg of tissues. The concentration of the sample was quantitatively determined by BCA protein assay. The lysate was mixed with loading buffer after heated at 100℃ for 5mins. The proteins were separated at 10%-15% SDS-PAGE colloid and then transferred to the PVDF membrane (Millipore, Germeny). Next PVDF membrane was seal with 5% non-fat milk for 1.5 hour and incubated with primary antibody at 4℃ overnight. Secondary antibodies (Antgene, Wuhan, China,1:10000) were used to incubate the membrane in shade environment at room temperature for 1h. The membranes were visualized with Odyssey (LI-COR biosciences, USA). The above show primary antibodies. Western blot analysis was repeated three times.

### Cell count kit-8(CCK8) assay

CCK8 assay, 3,000 glioma cells were resuspended in DMEM supplemented with 10% FBS and then added to a 96 well plate. Various concentrations of TMZ were added. Cell proliferation was investigated using CCK8 (Dojindo Molecular Technologies, USA) according to the manufacturer's instruction.

### TUNEL assay

In Fuorescein (FITC) Tunel cell apoptosis detection Kit (G1501, Servicebio, China) was used to detected DNA fragmentation in apoptotic cells according to the manufacturer's instruction. Only in apoptotic cells can there be green fluorescence localized by FITC-12-dUTP. Olympus BX51 microscope (Olympus, Japan) was used for image acquisition.

### Statistical analysis

Data were presented as mean values ± standard deviation (SD) from at least three experiments. Student's t-test was used to analyze the differences between two groups. Patients were divided into high and low groups according to the 50% cutoff point of SAA1 expression and Kaplan-Meier survival analysis was used to analyzed significance between groups. Pearson test was used for analyzing the correlation between SAA1 and other genes. Statistical analyses were performed using GraphPad Prism 8.0 software. A p value of less than 0.05 was considered as statistical significance.

## Results

### SAA1 is upregulated in human GBM and predicts poor prognosis

**Figure 1:**
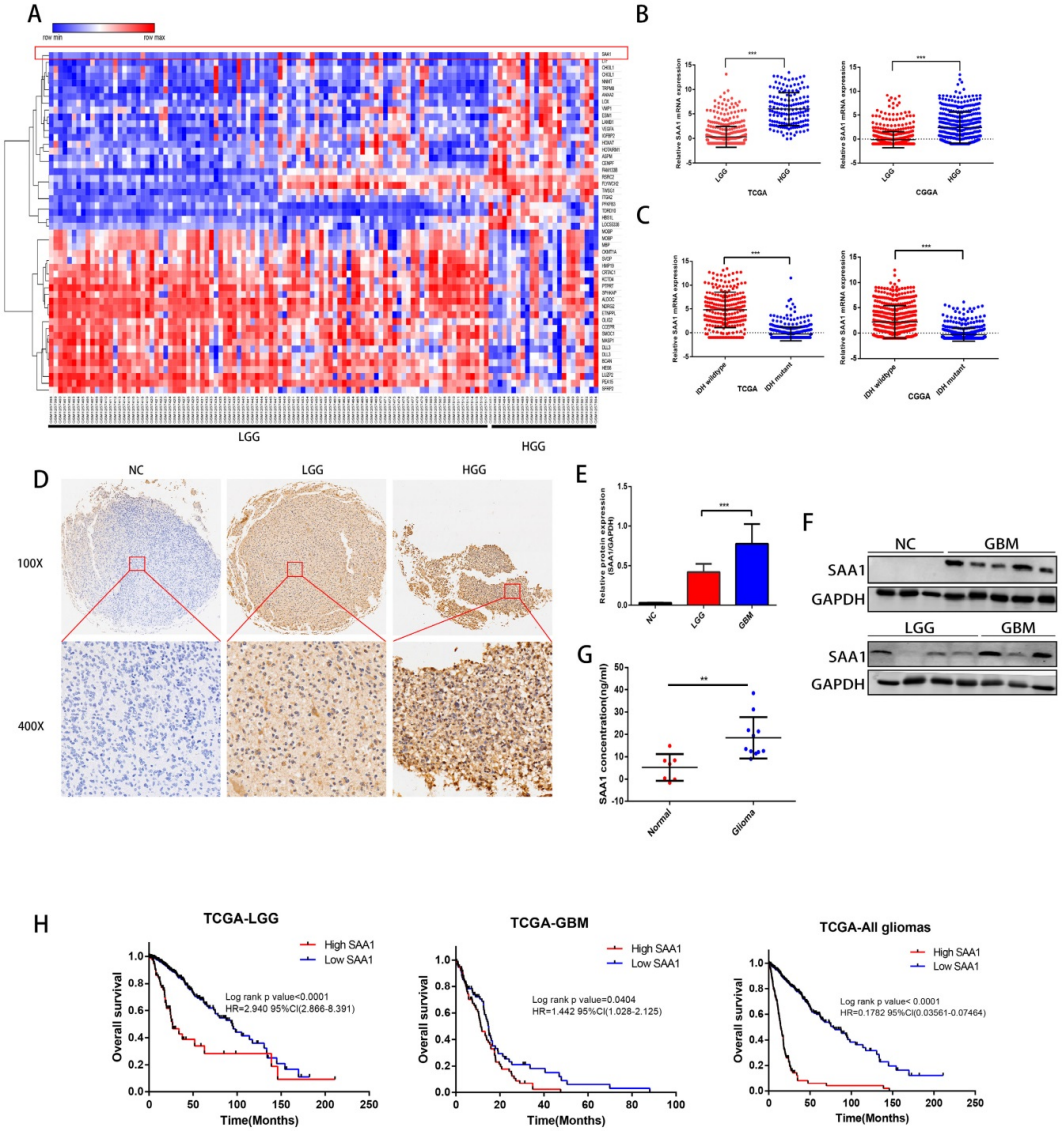
**SAA1 is upregulated in human GBM and predicts poor prognosis. (A)** The heat map of DEGs. The color gradient from red to blue represented the expression value (HGG/LGG) change from up-regulation to down-regulation. (|Fold Change|>2 and p value<0.01); **(B)** SAA1 mRNA expression in low grade glioma (LGG) and HGG in TCGA and CGGA datasets; **(C)** SAA1 mRNA expression in patients with wildtype and mutant IDH1/2 in TCGA and CGGA datasets; **(D)** Representative images of IHC staining of SAA1 in normal and glioma tissues; **(E, F)** Western blot was performed to compared SAA1 expression in gliomas and non-glioma, LGG, n=4; GBM, n=7; normal, n=3, GAPDH used as loading control; Images represented as the mean ±SD of three independent experiments; **(G)** The concentration of SAA1 protein in the serum of healthy and glioma patients, p value<0.01;** (H)** Kaplan-Meier survival analysis for SAA1 expression in all glioma patients in TCGA database, The median value of SAA1 levels was set as the cut-off level. HR, hazard ration. ***P<0.001.

We first performed differential expressed gene analysis based on 96 low-grade glioma samples and 24 GBM samples in the GSE52009 database, which visualize as a heat map (Fig. 1A). The expression of SAA1 in GBM is significantly higher than that of low-grade gliomas (logFC=2.06, p<0.0001). This result is also verified in the public TCGA and CGGA databases (Fig. 1B). And SAA1 was highly expressed in IDH wild-type patients (Fig. 1C). Moreover, SAA1 protein level was also elevated in HGG compared with that of LGG detected by IHC staining and WB (Figure 1D-1F). ELISA detected the concentration of SAA1 protein in the serum of 7 healthy or traumatic brain injury (TBI) subjects and 11 glioma patients. In the serum of patients with glioma, SAA1 levels are increased (Fig. 1G, p<0.01). Then we performed survival analysis according to data from TCGA database. The results indicate that patients with high SAA1 expression, regardless of low-grade gliomas or GBM, have a poor prognosis (Fig. 1H). These results verified that SAA1 was upregulated in GBM and predicted poor prognosis.

### SAA1 knockdown induces GBM cell apoptosis *in vitro*

**Figure 2:**
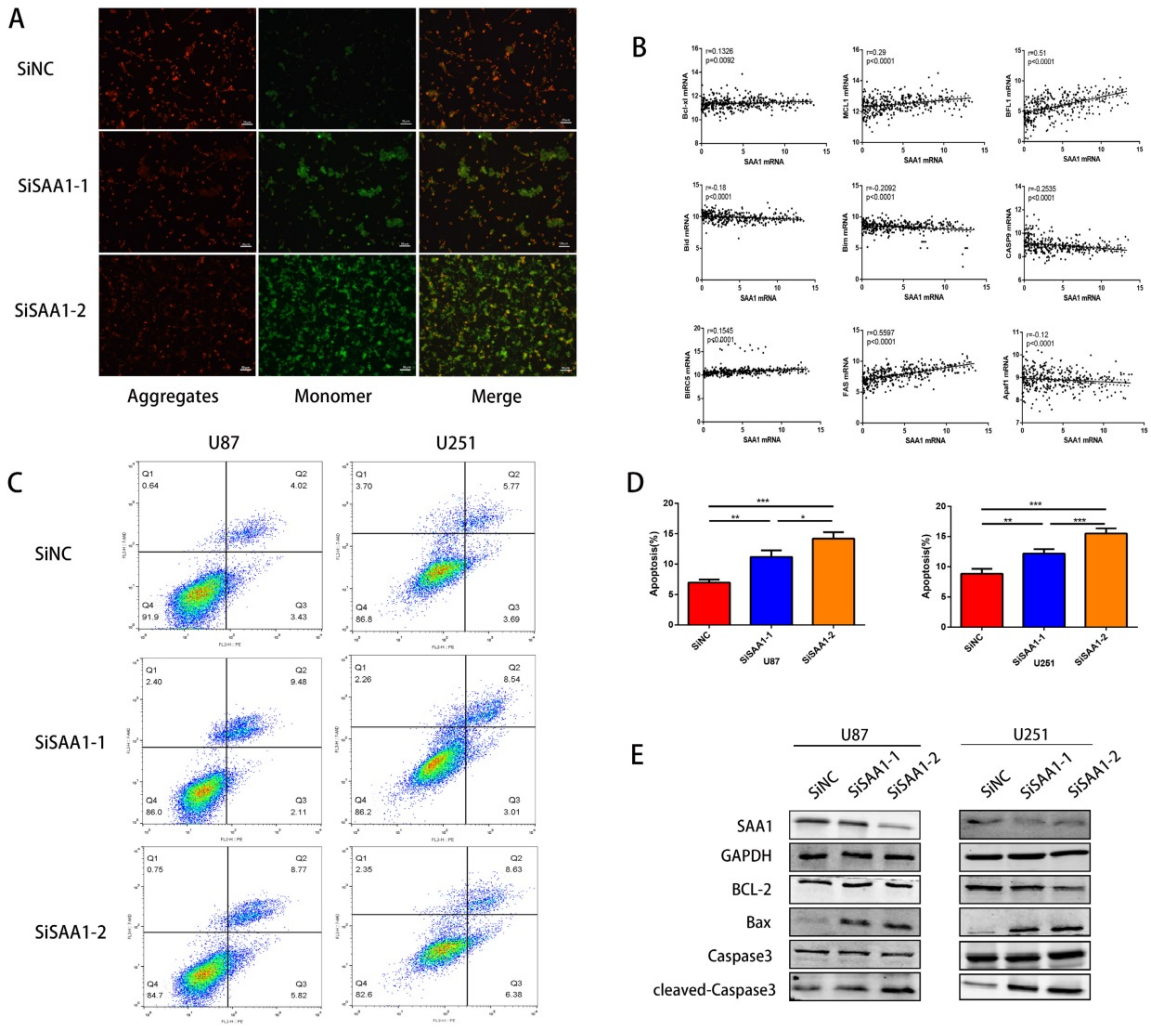
** SAA1 knockdown induces GBM cell apoptosis *in vitro*. (A)** ∆Ψm in U87cells according to JC-1 staining. A decrease in the ratio of red (aggregates)/green (monomers) fluorescence intensity indicates the loss of ∆Ψm. Scale bars, 50 µm; **(B)** The association between SAA1 and a series of apoptosis-related genes (Bcl-xl, BFL1, MCL1, BIRC5, Fas, Bim, Bid, CASP9 and Apaf1) in glioma patients was analyzed by the GlioVis platform. P-values were obtained from Pearson correlation; **(C, D)** U251 and U87 cells were transfected with negative control SiNC or siRNAs against SAA1 (siSAA1-1/2), followed by Annexin V-PE/7-AAD staining and flow cytometric analysis. **P<0.01, ***P<0.001; **(E)** Effects of SAA1 knockdown on the levels of apoptosis-related proteins in U87 and U251 cells. SiNC: negative control SiRNA. SiSAA1-1 and SiSAA1-2: two SiRNAs against SAA1.

To investigate the role of SAA1 in GBM, we performed loss of function study on U251 and U87 cells. Different SiRNAs that specific target SAA1 (SiSAA1-1 and SiSAA1-2) were synthesized and their knock-down effects were verified by western blot (first band in Fig. 2D). Firstly, we performed JC-1 staining to evaluate cell death. The decrease of mitochondrial membrane potential (∆Ψm) is a marker of early apoptosis, we found that SAA1 knockdown induced the loss of ∆Ψm in GBM cells (Fig. 2A). And the flow cytometry analysis of annexin PE / 7-AAD staining showed that SAA1 knockdown induced apoptosis in U87 and U251 cells (Fig. 2C). In addition, we found that in glioma tissues from the TCGA database, SAA1 levels were associated with a range of anti-apoptotic genes (Bcl-xl, BFL1, MCL1, BIRC5, and Fas) and pro-apoptotic genes (Bim, Bid, CASP9 and Apaf1) (Fig. 2B). To increase persuasion, we examined the expression of apoptosis-related proteins. Western blotting showed that SAA1 knockdown increased Bax and cleaved-caspase3 levels, decreased Bcl-2 levels, and total caspase3 levels remain almost unchanged (Fig. 2D). We found the expression level of SAA1 affected the apoptosis of glioma cells.

### SAA1 knockdown inhibits AKT phosphorylation

**Figure 3:**
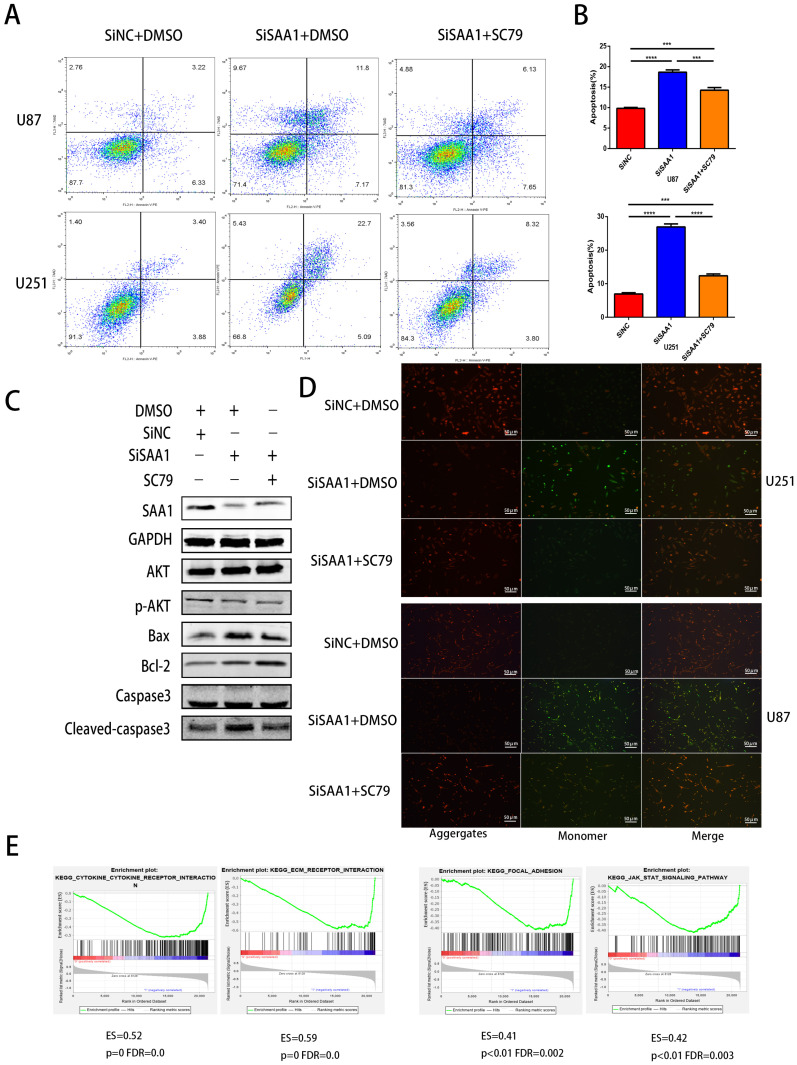
** SAA1 knockdown inhibits AKT phosphorylation. (A, B)** U251 and U87 cells transfected with SiNC or siSAA1-1 were treated with SC79(10µM) for 24h, followed by Annexin V-PE/7-AAD staining and flow cytometric analysis. Cell apoptosis was calculated by FACS. ***P<0.001; **(C)** U87 cells transfected with SiNC or siSAA1-1 were treated with SC79(10µM) or DMSO for 24h, Western blot was performed to compared levels of SAA1, AKT, pAKT and its downstream apoptosis-related proteins, GAPDH used as loading control; **(D)** ∆Ψm in U87 and U251 cells according to JC-1 staining. A decrease in the ratio of red (aggregates)/green (monomers) fluorescence intensity indicates the loss of ∆Ψm. Scale bars, 50 µm**. (E)** SAA1 regulates biology process associated with cytokine mediated signaling pathway, ECM-receptor interaction, Focal adhesion and Jak-STAT signaling pathway. ES: Enrichment Score, FDR: False discovery rate.

Previous studies 28 have found that many cytokines may activate AKT signaling pathways to effect phosphorylation of bcl-2 family members, thereby inhibite apoptosis and promote cell survival. Poor phosphorylation of AKT *in vitro* and *in vivo* blocks poorly induced primary neuronal death. Then we examine the expression levels of AKT, p-AKT and AKT pathway downstream apoptosis-related proteins. Our results revealed that SAA1 knockdown may promote apoptosis by decreasing p-AKT levels. Then, we used the AKT phosphorylation activator SC79 to perform cell experiments to eliminate the effect of SAA1 knockdown. The annexin PE/7-AAD and JC-1 staining results showed that after SC79 activator was added, the rate of apoptotic cells in U87 and U251 cells decreased then before, which was still higher than normal cells (Fig. 3A, B, D). Western Blot showed SC79 activator eliminates the effects of SAA1 down-regulation on the expression levels of BCl2, Bax and cleaved-caspase3 to varying degrees (Fig. 3C). At the same time, we found that AKT phosphorylation activator promoted the expression of SAA1, which indicated that there may be positive feedback regulation of SAA1 and AKT pathway.

There is no specific discussion on how SAA1 affects AKT phosphorylation, we conducted a GSEA study using glioma patient gene profiling data (GSE52009), and as shown (Fig. 3E), gene set differences in high versus low expression of SAA1 in GBM patients indicated that SAA1 may regulate biology process associated with cytokine-cytokine receptor interaction (p<0.01, ES=0.52), ECM-receptor interaction (p<0.01, ES=0.59), Focal adhesion (p<0.01, ES=0.41) and Jak-STAT signaling pathway (p<0.01, ES=0.42). SAA1 may affect the AKT pathway activation by affecting the above processes, or directly affect the AKT phosphorylation process, and further research is needed.

### SAA1 knockdown enhances the sensitivity of glioma cells to TMZ

**Figure 4:**
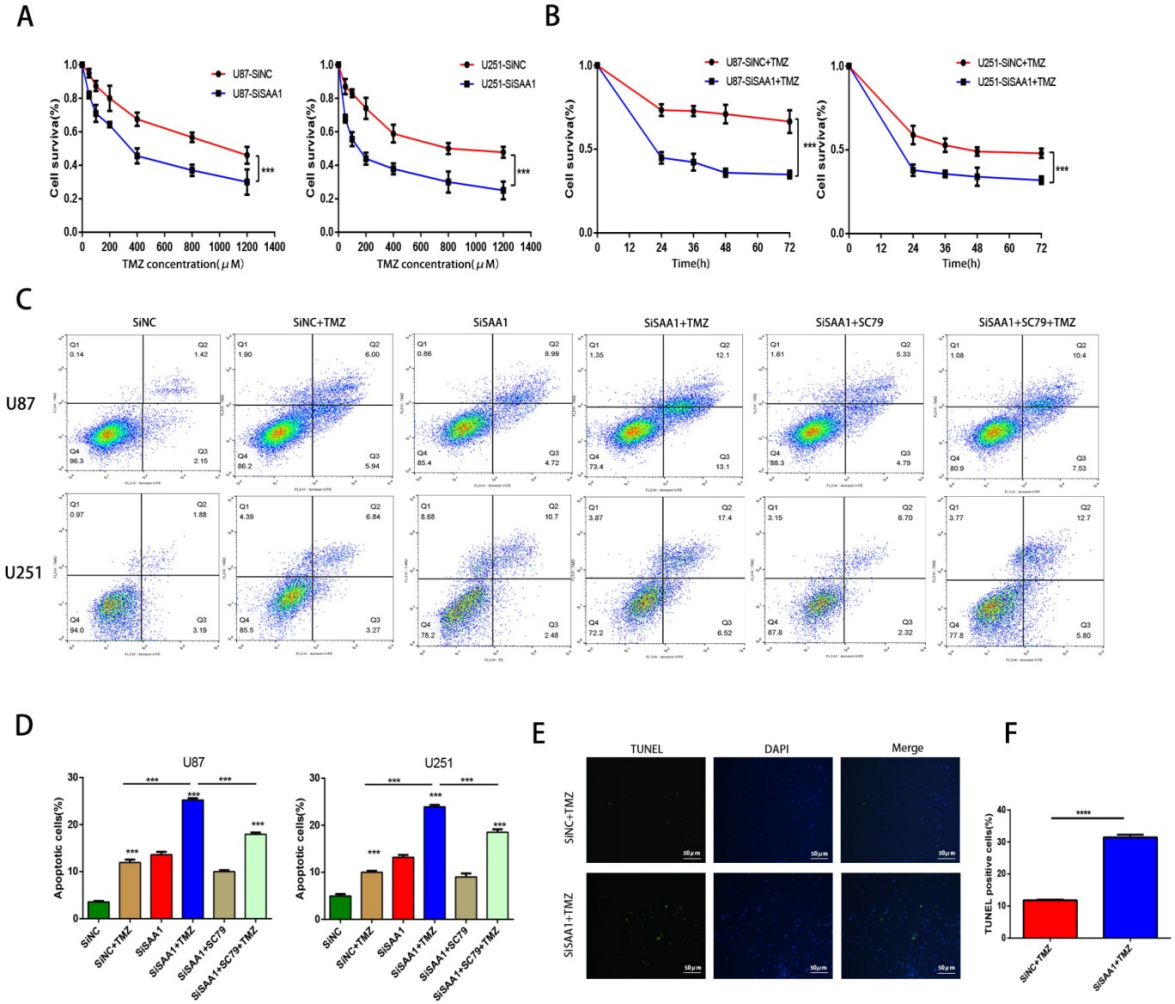
** SAA1 knockdown enhances the sensitivity of glioma cells to TMZ. (A, B)** CCK-8 assay was used to detect the survival rate of U87 and U251 cells treated with different concentrations of TMZ, and different times with certain TMZ concentrations (400μM);** (C, D)** U87 and U251 cells treated with certain TMZ concentrations (400μM). The apoptosis cells were detected by flow cytometry; **(E-F)** Representative images of TUNEL staining. Scale bars, 50 µm.

We used the CCK-8 assay to detect the survival rate of U87 and U251 cells treated with different concentrations of TMZ and the results showed that inhibition of SAA1 enhanced the efficacy of TMZ and decreased cell survival rate (Fig. 4A, B). The results of annexin PE/7-AAD revealed that SAA1 knockdown significantly increased GBM cells apoptosis caused by TMZ treatment, and AKT phosphorylation activator SC79 weaken the effects of SAA1 down-regulation (Fig. 4C, D). Our findings were verified by TUNEL assay and we found rate of TUNEL positive cells in SiSAA1 groups was much higher than that of SiNC group (Fig. 4E, F). These results strongly indicated that the down-regulation of SAA1 enhanced sensitivity to TMZ chemotherapy in GBM and might be a promising target. Taken together, we demonstrated that SAA1 knockdown promoted GBM cells apoptosis by regulating AKT signaling, which enhanced sensitivity to TMZ.

### SAA1 is a novel biomarker of response to TMZ in glioma

**Figure 5:**
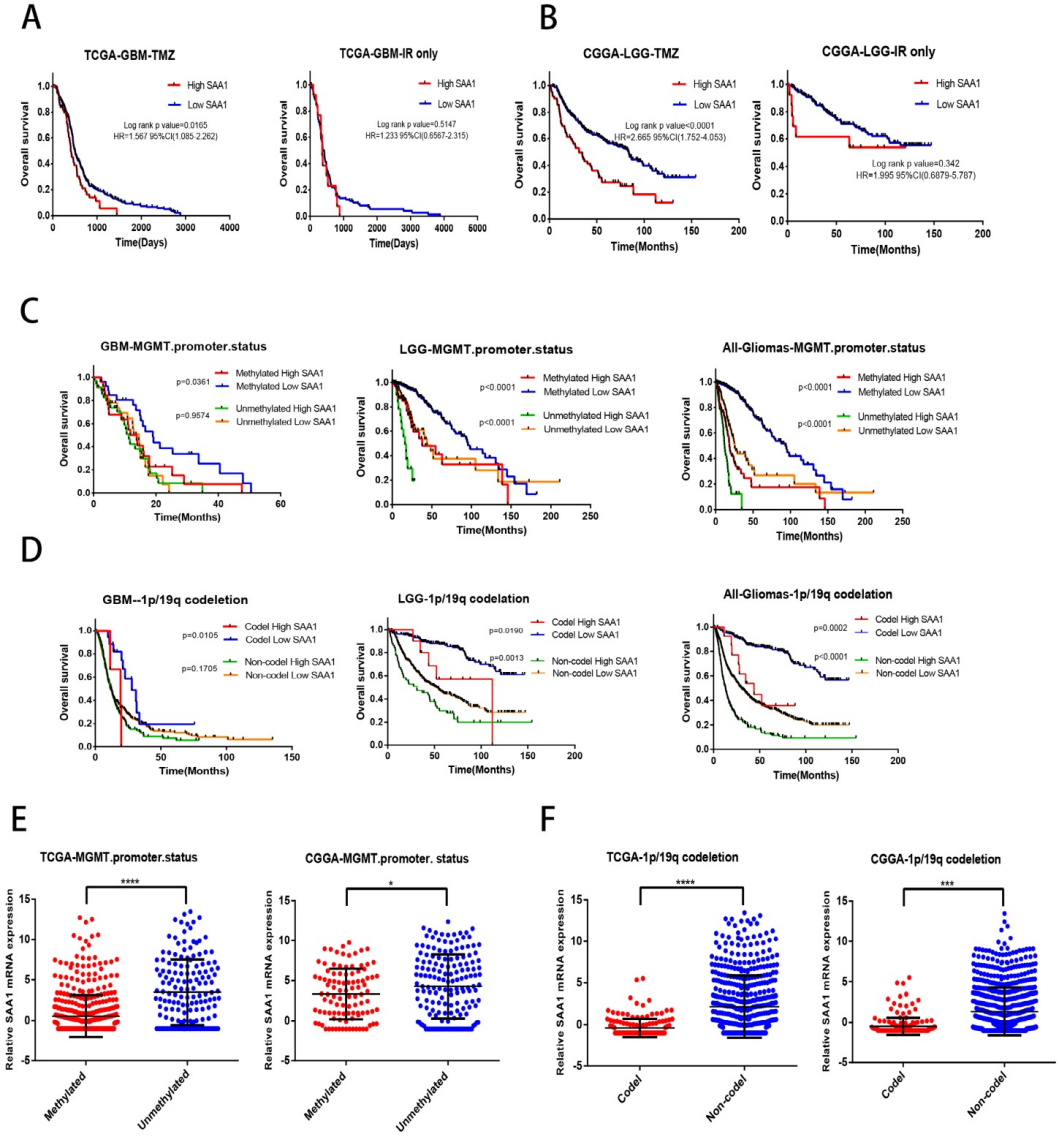
** SAA1 is a novel biomarker of response to TMZ in glioma. (A, B)** Kaplan-Meier overall survival curves in TCGA-GBM and CGGA-LGG patients. Patients were divided into groups according to median SAA1 expression and treatment modality (TMZ at any time vs IR only); **(C)** Overall survival in Gliomas. Patients were divided into groups according to median SAA1 expression and different methylation status of MGMT promoter. **(D)** Overall survival in Gliomas. Patients were divided into groups according to median SAA1 expression and different codeletion status of 1p/19q.** (E)** Relationship between SAA1 expression and MGMT methylation status in GBM.** (F)** Relationship between SAA1 expression and 1p/19q codeletion status in GBM. *p<0.05.

We found that SAA1 was highly expressed in IDH wild-type patients, and considering the relationship between IDH type and TMZ resistance, we tried to investigate whether SAA1 could be used as a biomarker of TMZ therapeutic-response. We analyzed the effect of SAA1 expression on the survival time of glioma patients with different treatments by using public TCGA and CGGA datasets. We found that GBM patients with low SAA1 expression survived significantly longer than those with high expression if they were treated with TMZ at any time, and the patients who were treated with ionizing radiation (IR) alone, SAA1 expression had no significant effect on survival time (Fig. 5A). Consistent with this finding, in LGG patients with high SAA1 expression had shorter survival time than those expressed low SAA1 if they treated with TMZ at any time, whereas SAA1 expression wasn't significantly associated with survival time of patients who were treated with IR alone (Fig. 5B).

In addition, MGMT methylation status and 1p/19q codeletion are often used to evaluate the sensitivity of patients to chemotherapy and radiation therapy in clinical. Then we further analyze whether SAA1 plays a role in it. First, patients with methylated MGMT promoter and 1p / 19q codeletion had lower SAA1 expression levels than those with unmethylated MGMT promoter and 1p / 19q non-codeletion (Fig. 5E, F). These results showed that SAA1 might be a crucial gene involved in chemoresistance of glioma to TMZ. Besides, in the GBM and LGG patients with methylated MGMT promoter, the lower the expression of SAA1, the longer the overall survival time. In LGG patients with unmethylated MGMT promoter, the same results were found, but there was no significant difference in GBM patients (Fig. 5C).

And patients with 1p/19q codeletion with low SAA1 expression had better prognosis, SAA1 expression had no significant effect on survival of 1p/19q non-codeletion GBM patients (Fig. 5D). Our data showed that patients with methylated MGMT promoter or 1p/19q codeletion who had high SAA1 expression presented similar prognosis with patients with unmethylated MGMT promoter or 1p/19q non-codeletion. Taken together, these results suggested that SAA1 was a novel biomarker in predicting TMZ response in glioma patients.

## Discussion

SAA1 is the precursor protein of inflammation-related amyloidosis. After different degrees of injury, infection, inflammation and new tumors occur in the body, its serum level will rapidly increase by more than 1000 times 30. High levels of SAA1 are associated with chronic inflammatory diseases, including atherosclerosis, rheumatoid arthritis and Alzheimer's disease (AD) 31. In brain diseases, study 32 has found that the abnormal expression of SAA in the AD brain may directly participate in the occurrence or continuation of the AD process. At the same time, more and more attention has been paid to the role of SAA1 in the occurrence and development of tumors. It is considered as a predictor of cancer risk and a prognostic marker, and plays an important role in the malignant development and prognosis of many tumors.

Previous studies have found that SAA1 is highly expressed in GBM 22. We verified the high expression of SAA1 in GBM through various databases such as GEO, TCGA, CGGA and clinical samples, and found that it predicts a poor prognosis. In this study, we found that SAA1 was upregulated in GBM and that SAA1 depletion induced GBM cell apoptosis *in vitro*. Mechanism studies reveal that SAA1 knockdown inhibits AKT phosphorylation to regulate expression of apoptosis-related proteins downstream of the AKT signaling pathway, induce apoptosis. AKT phosphorylation activator can significantly relieve the effect of SAA1 knockdown. At the same time, our results found that the down-regulation of SAA1 enhanced sensitivity to TMZ chemotherapy in GBM, and SAA1 was closely related to IDH type, MGMT promoter methylation status and 1p / 19q co-deletion status. SAA1 may was a novel biomarker in predicting TMZ response in glioma patients.

High levels of SAA1 are thought to be the cause of organ damage in diseases such as systemic amyloidosis and Alzheimer's disease. Studies have found that SAA has not only been shown to induce lysis of bacterial cells by forming ion channels in lipid bilayer membranes 33, but also to prevent cell death from eukaryotic cells 34, 35. Previous research has focused on the role of SAA1 in the transformation of malignant tumors, cell proliferation and anti-angiogenesis in uterine, lung, and nasopharyngeal carcinomas, and invasion and metastasis in gliomas 16, 17, 19, 22. Study 36 has found that the combination of SAA1 and integrin αvβ3 is involved in the occurrence and progression of glioma disease. Adhesion receptor integrin αvβ3 is considered to be a sign of angiogenesis, which initiates calcium-dependent signaling pathways, leads to endothelial cell migration, and plays an important role in vascular cell biology 37. SAA1 may regulate the proliferation and migration of endothelial cells induced by glioma by binding to integrin αvβ3, thereby promoting tumor angiogenesis. However, little is known about the effect of SAA1 on cell apoptosis in GBM. Our results show that SAA1 knockdown induces GBM cell apoptosis *in vitro*.

AKT plays a central role in the coordination of multiple signal transduction processes involved in transcriptional regulation, cell survival, and apoptosis. The main mechanism of AKT activation is the binding of ligands to cell membrane receptors. These ligands include growth factors such as insulin-like growth factor 1 (IGF-1) and platelet-derived growth factor (PDGF), cytokines, hormones, and mitogens 28, 38. Recently, it has been demonstrated that SAA may elicit cytokine and chemokine production and cell migration 39, 40. On the molecular level, SAA has been shown to stimulate several proinflammatory and anti-apoptotic signaling pathways including NF-KB, C/EBP, JNK, ERK, AKT and p38 to stimulate tissue remodeling and wound healing 41-43. Our study demonstrated that SAA1 may regulates GBM cells apoptosis by activating AKT signal pathway. We found that GBM cells reduced AKT phosphorylation after down-regulating SAA1 expression and increased apoptosis. The knockdown of SAA1 caused changes in the expression of BCL2 family proteins and apoptotic proteins such as Bax, Bim and Bid, as evidenced by Western blotting and high-throughput data analysis. In order to prove that the apoptosis of GBM cells caused by SAA1 knockdown is related to the decrease of AKT phosphorylation, we used AKT phosphorylation activator SC79 to eliminate the phosphorylation changes caused by SAA1 knockdown. Our results showed that SC79 significantly reduced GBM cell apoptosis after SAA1 knockdown. At the same time, we found that the expression of SAA1 increased to a certain extent after the increase of AKT phosphorylation. The specific mechanism is not clear. We speculate that there may be a positive-feedback regulation mechanism.

How SAA1 affects AKT phosphorylation is the direction of our next research. Some current studies may make conjectures. Studies have found that phosphoinositide 3-kinase (PI3K) recruits AKT to the plasma membrane to cause conformational changes, resulting in Thr308 site of AKT phosphorylated by phosphoinositide kinase 1 (PDK1) and Ser473 site phosphorylated by rapamycin complex 2 (mTORC2) 44, 45. In addition to mTORC2, there are many different kinases, such as dna-dependent protein kinase (DNA-PK), which is responsible for phosphorylation and activation of the Ser473 site of AKT 46. Tumor suppressor phosphatase and tensin homologues (PTEN) and PH domain leucine-rich protein phosphatase (PHLPP) negatively regulate AKT through dephosphorylation 47. In addition, GSEA results showed that SAA1 was likely to regulate biology process in GBM associated with cytokine-cytokine receptor interaction, ECM-receptor interaction, Focal adhesion and Jak-STAT signaling pathway. These signaling pathways are involved in cell adhesion, migration, proliferation, and apoptosis. SAA1 affects AKT phosphorylation through these pathways or other ways, and SAA1 may participate in more biological processes of GBM cells. There are vast research prospects here.

Besides its pivotal role in normal cellular physiology, many studies have demonstrated the activation of AKT cascade in various types of human cancer that often results in tumor aggressiveness and drug resistance 48. We found that the down-regulation of SAA1 enhanced sensitivity to TMZ chemotherapy in GBM. We used TCGA and CGGA datasets to analyze whether SAA1 expression affected survival of glioma patients who treated with different treatments. Our results demonstrated that SAA1 segregate both LGG and GBM patients into survival groups if they were treated TMZ at any time, which gave us a hint that SAA1 might be related to TMZ resistance. MGMT methylation level and 1p/19q codeletion is an important indicator to predict the effect of TMZ-chemotherapy. Our data showed that in patients with methylated MGMT and 1p/19q codeletion who had high SAA1 expression presented similar prognosis with patients with unmethylated MGMT or 1p/19q non-codeletion. Among those patients, patients with methylated MGMT or 1p/19q codeletion that also had low SAA1 expression had best prognosis. These results indicated that SAA1 may was a novel biomarker of response to TMZ in glioma.

In summary, SAA1 may affect GBM cell apoptosis by regulating AKT phosphorylation. Down-regulation of SAA1 can inhibit AKT phosphorylation to affect downstream signaling proteins, cause GBM cell apoptosis. At the same time, SAA1 may be related to TMZ sensitivity. Hence, SAA1 might be a potential therapeutic target and prognostic factor for treating GBM.

## Abbreviations

SAA1: Serum amyloid A1
GBM: glioblastoma
AKT: serine/threonine protein kinase B
TMZ: Temozolomide
FPRL1: formyl peptide receptor-like-1
NO: nitric oxide
MMPs: metalloproteinases
BAD: bcl-2 cell death antagonist
GEO: Gene Expression Omnibus
TCGA: The Cancer Genome Atlas
CGGA: Chinese Glioma Genome Atlas
GSEA: Gene set enrichment analysis
CST: Cell signaling Technology
IGF-1: insulin-like growth factor 1
PDGF: platelet-derived growth factor
PI3K: phosphoinositide 3-kinase
PDK1: phosphoinositide kinase 1
mTORC2: rapamycin complex 2
DNA-PK: dna-dependent protein kinase
PTEN: phosphatase and tensin homologues
PHLPP: PH domain leucine-rich protein phosphatase
AD: Alzheimer's disease
